# What do we know about limiting after-hours availability expectations and work-related connectivity? A systematic review of interventions and policies

**DOI:** 10.5271/sjweh.4277

**Published:** 2026-05-01

**Authors:** Wendy Nilsen, Tanja Nordberg, Kristine Lescoeur, Mari Holm Ingelsrud, Cathrine Egeland

**Affiliations:** 1 Work Research Institute, OsloMet – Oslo Metropolitan University, Oslo, Norway.

**Keywords:** boundary management, detachment, disconnection, health, long work hour, off-job work, right-to-disconnect, technology-assisted supplementary work, well-being

## Abstract

**Objectives:**

Concerns about the health and well-being effects of high after-hours availability expectations and work-related connectivity have prompted calls for organizational and national disconnection measures, such as the right-to-disconnect legislation. However, the effectiveness of such measures remains unclear. This is the first systematic review that aims to evaluate interventions and policies designed to limit availability expectations and after-hours work connectivity.

**Methods:**

We searched Embase, Medline, PsycINFO, and Web of Science for studies published (2004-2024) for peer-reviewed empirical studies. Two reviewers independently screened records extracted data and assessed study quality using the Effective Public Health Practice Project tool. The review was registered in PROSPERO (CRD42024599491). Effectiveness was assessed using a structured qualitative approach that accounted for various study design and methodological rigor across intervention types.

**Results:**

Twelve studies (N=2306) were included: one national policy, three organizational disconnection guidelines, one supervisor-targeted program, and seven employee-focused programs. Half of the quantitative studies were randomized controlled trials; overall methodological quality was rated as weak. Most organizational and national-level policies showed limited or no effects, with benefits contingent on the person–environment fit and implementation quality. Supervisor-targeted and multi-component programs, particularly those allowing for flexibility and combining boundary management with other elements, showed significant modest effects on detachment, boundary control, and work–life balance, though effect sizes were generally small.

**Conclusions:**

The evidence base is small, heterogenous, and methodologically limited. Policies alone are unlikely to reduce harmful connectivity without active organizational implementation and cultural change. Developing and testing rigorous, multi-level interventions that address norms and supervisory practices, as well as individual boundary preferences, are urgently needed.

Increasing proportions of workers are connected to their workplace outside working hours. Findings among European employees (ie, Belgium, France, Italy and Spain) with the possibility to work remotely show that in a typical work week, 67% were contacted by their manager outside contractual work hours some days or more (via e-mail or phone) (1). Even more, 74% were contacted by their colleagues outside contractual work hours some days or more (1). As advancements in technology allow work to creep into the private sphere, there is a growing concern about employees' lack of recovery. Research over the past decade consistently shows that “always on” availability expectations (2) and work-related connectivity outside work hours (3) carry significant costs for employees' well-being and work–life balance. These concerns have led to a call for guidelines, policies or other preventive efforts that can protect employees against adverse consequences, for instance by reducing availability demands and the pressure to be connected outside work hours (4). There have also been calls for stronger regulations of availability expectations and work-related connectivity such as “the right to disconnect” (5, 6). Still, not much is known about which interventions (eg, policies, guidelines, mitigation efforts) can efficiently limit the pressures of being available for and connected to work outside work hours (7). The objective of the current study was thus to systematically map and review studies examining the effects of interventions and policies aimed at limiting availability expectations and work-related connectivity outside work hours.

## After-hours availability expectations and work-related connectivity

Based on former work, we define *after-hours availability expectations* as the perceived expectation that employees should remain reachable and responsive to work-related communications, such as emails, calls, or messages, outside of contracted working hours (8). After-hours availability expectations can stem from external expectations and explicit requirements from colleagues and supervisors in the organization (9) but can also be implicit, stemming from contextual cues such as workplace norms, values, perceived obligations and professional culture (10). Findings show that availability expectations are negatively associated with employee's health and work outcomes, such as occupational stress, work–life conflict and emotional exhaustion in cross-sectional and longitudinal studies (2, 4, 11). The association between availability expectations and adverse outcomes have been found to also depend on work demands. For instance, Dettmers et al (8) showed that beyond the relationship between availability expectations and recovery outcomes, experiencing a high degree of after-hours work-related communication was important. When employees experience high levels of availability expectations, they are also more likely to engage in after-hours work-related connectivity (12).

*After-hours work-related connectivity* refers to employees' often unpaid engagement in work tasks or communication (eg, emails, messages, calls) outside of regular working hours, facilitated by digital devices such as smartphones or laptops (13–15). Although after-hours connectivity has benefits (eg, increased autonomy to meet work and family demands and job satisfaction), several studies indicate that extensive after-hours work connectivity has adverse consequences for employee's health and well-being, such as decreasing recovery and psychological detachment and increasing work–life conflict (3, 4, 16), and with consequences spilling over to marital satisfaction and partner's stress levels (17). Availability expectations and after-hours connectivity are related constructs. The former refers to a “perceived obligation”, while the latter may reflect the behavioral response to that obligation. For instance, Mellner et al (4) stated that connectivity represents a “harmful response” to availability expectations. Due to this close association supported by former findings (eg 13,), we have chosen to include both as the focus in the current review.

Work environments characterized by high levels of work-related connectivity are also often characterized by high temporal and spatial flexibility. Taken together, the increasing flexibility of modern work highlights a need for organizations to promote healthy norms around after-hours availability expectations and work-related connectivity. Without such efforts, employees may face increased risks of burnout and stress-related illness due to the always-on culture, driving up sickness absence and turnover intentions (11, 13). These outcomes have significant implications for both employee well-being and organizational performance. Still, the responsibility for managing work outside regular hours often falls predominantly on the individual employee rather than the organization (18, 19). While some workplaces have introduced guidelines and policies to prevent work-related matters from entering nonwork life, such as advising against or restricting access to after-hours communication, there are often no clear guidelines in place. Employees are thus often left to craft their own solutions which might vary widely in effectiveness (18). Research indicates that employees frequently respond to work-related interruptions, with maladaptive coping mechanisms (20). This underscores the need for evidence-based knowledge that organizations can use to provide clear guidelines or other efforts that can mitigate the adverse effects of harmful availability expectations and thus promote sustainable well-being at work.

## Right-to-disconnect policies on national level

Several countries, such as France, Belgium, Spain, Italy and Australia, have now passed national legislation establishing a “right to disconnect” to protect employees from being expected to engage in work-related communication outside regular working hours (1, 21). Comparative legal analyses show significant variation in how countries define and implement the right, shaped by differing labor market regimes and collective bargaining traditions (eg 22, 23,). In the first country to pass a formal right to disconnect (France in 2017), the legislation mandates that all companies with ≥50 employees must implement mechanisms to regulate after-hours digital communication through negotiations with the unions. The legislation states that a collective agreement in the workplace should be established; if that is not possible, the company must adopt a charter of good conduct. However, it is uncertain if the legislation in France and other countries that followed its lead has had an actual impact on availability expectations and after-hours connectivity patterns or employees' well-being.

## The current study

Despite increasing concerns and findings about the adverse consequences of extended work availability, and the implementations of right-to-disconnect policies across several countries and organizations, limited evidence exists on which interventions or preventive efforts (eg, policies, guidelines) efficiently reduce unhealthy availability expectations and work-related connectivity outside work hours.

Previous reviews of related constructs have focused on the predictors and consequences of psychological detachment (24, 25) and general technostress (26). Some reviews have mapped interventions promoting psychological detachment in general (27), but to our knowledge no study have synthesized interventions specifically targeting availability expectations or after-hours connectivity. This review extends prior work by integrating evidence across individual, organizational and policy levels, focusing explicitly on interventions designed to manage connectivity rather than broader well-being, psychological detachment or general challenges with technology. The aim in the current review was therefore to systematically map and evaluate quantitative and qualitative empirical studies to answer the following research questions: (i) which types (eg, policies, guidelines) of interventions or preventive efforts have been implemented to reduce availability expectations and work-related connectivity in employees?; (ii) at which levels (eg, individual, organizational, national), and with which study designs have these efforts been evaluated?; and (iii) to which degree do these interventions improve individual, familial and work-related consequences?

## Methods

### Search and screening strategy

Three steps were used to identify relevant studies. First, a systematic search was conducted in the electronic databases: Embase, Medline, PsycINFO and Web of Science. We used three clusters of keywords and subject headings related to the (i) Outcome: availability expectations and work-related connectivity outside work hours (eg, constant availability, technology-assisted supplemental work, after-hours work, always-on, availability expectations); (ii) Context: the workplace (eg, employee, organization, business); and (iii) Intervention (eg, trial, intervention, RCT, experiment, evaluation, guideline, policy, tactics). See the supplementary material (https://www.sjweh.fi/article/4277, table S1) for the search strings. The search was limited to English-language studies published in the past 20 years (2004–2024). The reference list and citations of the included studies were checked to find further relevant studies. The systematic review is registered within the Prospective Register of Systematic Reviews (PROSPERO) (https://www.crd.york.ac.uk/PROSPERO/view/CRD42024599491). Guidelines of the Preferred Reporting Items for Systematic Reviews and Meta-Analyses Protocols Checklist (28) were used.

Second, the citations were uploaded to an open-source AI application, ASReview (29), designed to systematically screen and select studies. We pre-screened and labeled 100 studies as relevant or irrelevant to train ASReview. The application learns from the reviewer's feedback and uses this knowledge to intelligently sort the publications by relevance and present assumed relevant publications for review, with machine learning. We screened the AI-selected studies until 370 records had been labelled (21.5% of the data set), at least twice the number of estimated relevant records had been screened, and only one relevant record had been identified in the last 150 records. Third, two review authors, independent of each other, retrieved and read the full text of the relevant studies for inclusion or exclusion. When uncertain, inclusion was discussed among the reviewers.

### Inclusion and exclusion criteria

Studies with an empirical study were included when they were peer-reviewed and met all the following criteria: (i) participants were employed or self-employed adults; (ii) examined an intervention or preventive effort, including policies and guidelines aimed at reducing availability expectations and/or work-related connectivity outside work hours; and (iii) comprised any individual (eg, well-being, health, burnout and pain), familial (eg, partner relationship, time with family, work-family conflict) or work-related (eg, sickness absence, productivity, concentration at work) outcome. Studies with and without control groups were included.

We excluded “grey literature”, eg, reports, books and conference proceedings that were not peer-reviewed, as well as non-empirical studies, eg, theoretical papers and essays to ensure quality and comparability. Legal doctrinal analyses where not a part of the study as they did not include employees or employers as participants. Of note, self-employed workers in addition to organizational employed workers were included in this review. Although they operate under different organizational and regulatory conditions than organizationally employed workers, individual-level boundary management interventions are applicable across employment arrangements, including self-employed workers. We note, however, that the proportion of self-employed participants in the included studies was small and does not materially affect the interpretations of results.

### Data extraction

The data were extracted using a pre-defined standardized form covering: (i) publication factors (authors, publication year, journal and title of the study); (ii) sample description (country, sample size and profession); (iii) aim and study design (method; comparator/control; outcome measures); (iv) intervention factors (description, target and level of intervention); and (v) outcomes and results. One reviewer extracted data from the individual studies, which a second reviewer then checked. If the same studies reported multiple measures of the same outcome, we prioritized validated scales over single-item instruments.

### Quality assessment and evidence synthesis

Two independent reviewers assessed the quality of each study. Discrepancies were resolved by involving a third reviewer. Quantitative studies were assessed with the Effective Public Health Practice Project (EPHPP) checklist (30). The EPHPP assesses selection bias; study design; confounders; blinding; data collection methods, and withdrawal and drop-outs. Studies were rated on each component from weak (score of 1) to strong (score of 3) (30). Global ratings were assigned according to the EPHPP guidelines; high quality = no weak ratings; moderate quality = one weak rating; low quality = two or more weak ratings (29). Studies were not excluded based on a low score.

Qualitative studies were assessed with the Joanna Briggs Institute (JBI) Critical Appraisal Checklist for Qualitative Research, which consists of ten items assessing aspects such as congruity between philosophy, methodology, methods and interpretation; representation of participants' voices; ethical approval; and researcher reflexivity. We conducted a narrative synthesis of the findings grouped after the level of the interventions; national, organizational and individual level.

## Results

**Figure 1:**
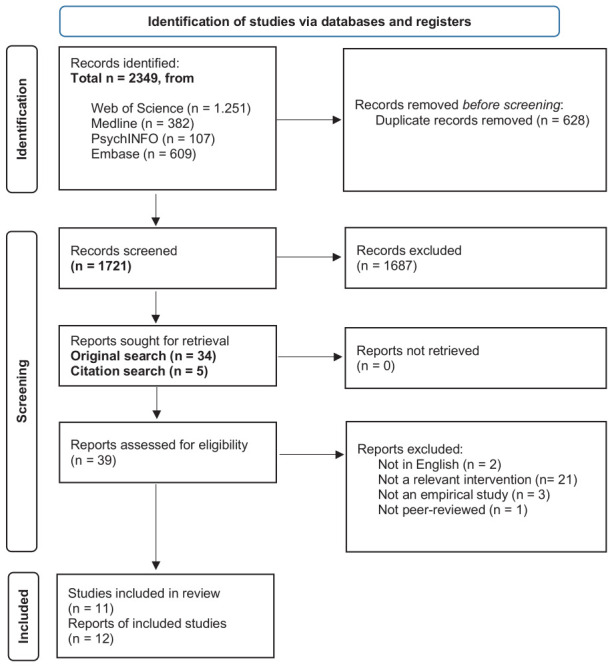
Prisma flow chart. Source: Page MJ, et al. BMJ 2021;372:n71. doi: 10.1136/bmj.n71.

The search revealed 1721 titles after removing duplicates (see supplementary table S1 for in-depth search strings and findings per data base). In total, 34 titles matched the inclusion criteria from the search, and five titles were added after citation check and through authors' libraries. Two titles and abstracts were in German and were not retrieved due to the language restriction. In total, 32 titles were retrieved, and read in full text by two reviewers. A total of 12 publications met the criteria and were included. See the PRISMA flow chart in figure 1.

The 12 included publications (2018–2024) evaluated a range of interventions across three levels: national policy (N=1), organizational guidelines (N= 3), and employee- or supervisor-focused programs (N=8). Most were conducted in Germany (N=8), with others from Sweden, France, the UK and the US. Study designs included randomized controlled trials (RCT) (N=5), pre- and post-test trials (N=2), quasi-experiments (N=1), observational survey studies (N=2) and qualitative interviews (N=2). Few studies directly assessed changes in availability expectations and after-hours connectivity, and effect sizes were often not reported. See table 1 for national- and organizational level policies and table 2 for individual-level interventions.

**Table 1:** Overview of studies evaluating policies and guidelines on the organizational level

Author	Sample	Aim of the study	Design	Policy	Outcomes	Results
Barber et al, 2023	235 employees from various professions in the US (sample 1); and 482 employees from various professions in the US (sample 2)	Examine if having disconnection policies versus norms (availability expectations/family supportive environment) in the workplace is associated with telepressure.	Cross-sectional survey	Presence of a disconnection policy in the workplace	Telepressure	Disconnection policies in the workplace were not significantly associated with telepressure in either sample. Implicit norms, availability expectations and family-supportive environment was associated with telepressure.
Mueller & Kempen, 2023 (Study 2)	239 employees from various professions in Germany	Examine the influence of employee boundary management preference on the policy effectiveness of off-hours work-related communication restrictions	Cross-sectional survey	Self-reported communication restrictions in the workplace (0-100 scale)	Boundary management preferences, person-environment fit (i.e., boundary management preferences-policy fit), policy satisfaction, organizational commitment, emotional exhaustion, and work engagement	Communication restrictions in the workplace were associated with improved policy satisfaction, organizational commitment and emotional exhaustion, for employees who prefer to segregate work and family, through high employee-policy fit. Communication restrictions had a negative direct effect on work engagement.
Pansu, 2018	20 employees and managers (interviews) and 107 employees (surveys) from various professions in France	Examine the impact of the “Right to Disconnect” legislation in France on workers’ productivity and examine perceptions about the legislation	Semi-structured interviews and cross-sectional survey	The “Right to Disconnect” national legislation in France, which posits that all companies with 50 employees or more are required by law to regulate the use of digital tools, either through a collective agreement or employer-created charter of good conduct	Productivity and perceptions about the legislation	Most employees (97%) report no observable changes in their organizations. Employees support the right to disconnect and expect work–life gains. Barriers to implementations are minimal managerial engagement, lack of enforcement and focus on productivity and heavy workload (which is accomplished due to after-hours work). Impact of the legislation is therefore seen as modest, and consequences for productivity as insignificant.
Reis, 2024	30 employees (23 IT workers and 7 of their managers) in the German production industry	Examine which organizational mitigation measures have been implemented to reduce techno-invasion and techno-overload, and how employees and managers experience these mitigation measures	Qualitative interviews	Technological, cultural and social measures to reduce techno-invasion (i.e., the feeling that technology enables work to intrude into personal life) and techno-overload (i.e., the experiences of being digitally overwhelmed)	Types of mitigation methods, mitigation efficacy and unintended consequences	They reported eight organizational efforts: separating private and business devices, restricting email traffic, introducing emergency-only communication channels, promoting clear availability expectations around non-work time, and improving internal ICT practices (e.g., less emails). The measures were seen to reduce interruptions, unnecessary information, and workload, improve work–life balance and availability clarity. Managers had concerns about reduced organizational efficiency and flexibility, and employees reported unintended consequences, such as increased message overload during work hours.

**Table 2:** Overview of studies evaluating individual-level interventions

Author	Sample	Aim	Design	Intervention	Outcomes	Results
Althammer et al, 2023	373 employees from various professions in Germany (web-based = 107; blended = 129; waitlist control = 137)	Examine the effect of the “FlexAbility”-intervention, aimed to improve coping with challenges with flexible work designs (telework or flextime), with positive activities	Randomized controlled trial with 4-week and 6-month follow-up	A 6-week web-based intervention to improve coping with flexible work challenges. The intervention involved a multi-component self-regulation toolkit of positive activities/strategies considering segmentation, mindfulness, self-organization, and recovery. The web-based group had only individual sessions (6×45 min), while the blended group also had 3 online group sessions. The participants were encouraged to practice the activities most fitting their own needs and preferences, in line with person-activity fit perspectives.	Psychological detachment, satisfaction with work-life balance, positive affective well-being	The intervention was effective, with significant improvements in psychological detachment, work-life balance and positive affective well-being, in both intervention groups versus the waitlist group, from baseline (T0) to after the intervention (T1) and 4-weeks after (T2). Additionally, significant improvements for psychological detachment were found to last six months after (T3).
Althammer et al, 2024	288 employees from various professions in Germany (intervention = 105; control = 183)	Same as above	Randomized controlled trial with 4-week follow-up	A 6-week web-based intervention to improve coping with flexible work challenges. The intervention involved a multi-component self-regulation toolkit of positive activities/strategies considering segmentation, mindfulness, self-organization, and recovery, with 45-min sessions each week. The participants were encouraged to practice the activities most fitting their own needs and preferences, in line with person-activity fit perspectives.	Emotional exhaustion, satisfaction with work-life balance, positive emotions (mediator), and boundary management (mediator)	The intervention was effective, with significant improvements in emotional exhaustion, work-life balance satisfaction, positive emotions and boundary management, in the intervention versus the control group, from baseline (T1) to after the intervention (T2) and 4-weeks after (T3). Effect sizes were small.
Althammer et al, 2025 (Note: Same data as Althammer et al, 2023)	358 employees from various professions in Germany (intervention = 147; control = 211)	Same as above	Randomized controlled trial with 4-week and 6-month follow-up	Same as above	Psychological detachment, strain-based work-family conflict, positive affective well-being, stress, work engagement, and self-regulation (mediator)	The intervention was effective with significant improvements in strain-based work-family conflict, positive affect, stress and work engagements in the intervention versus the control group, from baseline (T1) to after the intervention (T2). The effects sizes were small. Additionally, improvements for psychological detachment, strain-based work-family conflict and positive affect were found to persist at 4-week follow-up (T3), with small to medium effect sizes. This effect persisted to the 6-month follow-up (T4)
Edvinsson et al, 2025	167 governmental office workers in Sweden (intervention = 97; control = 70)	Examine the effect of a workplace intervention, aimed at promoting recovery in office workers with flexible work designs	Quasi-experimental design with 6- and 12-month follow-up	A two-step workplace intervention to promote recovery among office workers with flexible work arrangements. The program combined 1) A four-month individual-level course (three sessions) providing strategies to work more efficiently with ICT and manage boundaries between work and non-work time, and 2) A 6-hour workgroup-seminar where teams co-developed common rules and routines regarding availability expectations and work-nonwork boundaries.	Need for recovery, psychological detachment	The intervention had no significant direct effect on need for recovery or psychological detachment across the 6- and 12-month follow-up periods, but the intervention was effective in recovering employees with high need for recovery.
Heissler et al, 2024 (Study 2)	23 supervisors and 62 of their team members in a German transportation company	Examine the effect of a training intervention for supervisors, aimed at reducing availability expectations and ambiguity	Pre- and post-test study with 6-week follow-up	A half-day supervisor workshop providing evidence-based guidance on the consequences of extended availability and after-hours ICT use, and equipping supervisors to set and communicate clear availability expectations, prevent unnecessary and unintended ICT use and availability after hours, and establish explicit after-hours availability agreements.	Employee-reports: Availability agreement, expectations and ambiguity, satisfaction with ICT-communication, detachment, emotional exhaustion and work-life balance	The intervention was effective in significantly increasing explicit availability agreements and decreasing availability ambiguity reported by team members from baseline to six weeks after the intervention. The intervention was not effective in significantly improving availability expectations, satisfaction with ICT-communication, detachment, emotional exhaustion or work-life balance. Supervisors reported being satisfied with the workshops and rated the intervention as useful.
Pfaffinger et al, 2023	50 employees (informational intervention = 22; control group = 28)	Examine the effect of an informational intervention, aimed at reducing digitalization-related stress in employees	Randomized controlled trial with 2-week follow-up	A 12-day app-based intervention to reduce digitalization-related stress. Participants received an informational intervention providing daily advice on managing ICT-related demands (i.e., the benefits of using “do not disturb”-features during leisure time or not checking work-related messages before 8.00 in the morning). Interventions lasted 10-15 min per session.	General well-being (stress and strain), work engagement, job satisfaction, detachment, technostress, digitalization anxiety, and IT-resilience	The information intervention had adverse effect, by increasing general stress in the intervention versus the control group, across the 2-week follow-up period. The intervention had no significant effect on any other outcome.
Reinke, 2024	75 employees from various professions in Germany (intervention = 31; control = 44)	Examine the effect of the “Switch off and recharge” boundary management intervention	Randomized controlled trial with 1-month follow-up	A 4-week web-based intervention (4×45-60 min) targeting boundary management. The intervention included psychoeducation, practical exercises for behavior change (e.g. goals and action plans), learning boundary tactics, and how to use this in their daily lives in the long run.	Perceived learning, boundary control, boundary creation, psychological detachment, satisfaction with work-life balance, positive and negative affect	The intervention was effective with significant improvements in work-related ICT use, perceived learning, boundary control and enactment, psychological detachment, satisfaction with work-life balance and negative affect, in the intervention versus the control group, from baseline to 1 month after the intervention. There was no significant effect for positive affect.
Rich et al, 2020	22 hospital doctors-in-training in the UK	Examine the effect, feasibility and acceptability of the iWARDS-intervention for doctors-in-training	Pre- and post-test study, with 1 month follow-up, and interviews	A series of six face-to-face workshops (6×120 min) to promote general and digital well-being. The program combined self-care strategies (e.g., healthy eating/sleep, mindfulness, and self-compassion) with microboundary techniques to manage technology use and improve work–life balance (e.g., notification control, availability expectations). The workshops included group discussions, reflective exercises, and setting SMART goals to support implementation.	Burnout, well-being, boundary control	The intervention was effective with significant improvements in burnout and boundary control, but not well-being, after 1 month. Self-care and microboundary strategies were highly valued, and experienced as improving participants’ awareness, well-being, and ability to set work–nonwork boundaries. However, heavy workloads, rotating shifts, and fear of missing out on work or personal matters limited implementation.

### National level policy

Only one study examined a national right-to-disconnect policy. Pansu (31) evaluated the French legislation two years after its implementation using a combination of semi-structured interviews and a short survey study. The study reported a limited perceived impact: 97% of surveyed employees reported no observable changes in how after-hours communication was managed in their workplace. The interviews underscored key implementation barriers, including minimal managerial engagement, lack of enforcement, and the absence of workplace-level follow-up. While the French legislation requires organizations (with ≥50 employees) to establish agreements or charters regulating after-hours digital communication, the study indicates that many employers take a passive or symbolic approach, resulting in little change in actual norms and practices.

### Organizational level disconnection policies and practices

Three studies evaluated the effects of workplace-level disconnection policies. Two quantitative studies (32, 33) found no direct improvements in employee well-being, availability expectations (ie, telepressure) or occupational outcomes. Barber et al (32) reported that employees in organizations with disconnection policies experienced similar levels of digital pressure as those without, likely reflecting the fact that 39–64% reported that the policies were not actively enforced. Similarly, Mueller & Kempen (33) found no direct impact of disconnection policies on employee outcomes but found indirect moderated benefits, such as reduced emotional exhaustion and higher organizational commitment for employees who preferred clear work-nonwork boundaries when the person-policy fit was high. These findings suggest that disconnection policies may be more effective when aligned with individual preferences.

Complementing these findings, a qualitative study by Reis et al (34) reported eight organizational efforts to reduce techno-invasion (ie, the feeling that technology enables work to intrude into personal life) and techno-overload (ie, the experiences of being digitally overwhelmed). Key measures included separating private and business devices, restricting email traffic, introducing emergency-only communication channels, promoting clear availability expectations, and improving internal ICT practices (eg, pull-not-push culture with less emails). Participants generally valued disconnection strategies (eg, email restrictions, the promotion of disconnecting) for helping set clearer boundaries, but no measure was seen as only beneficial. Managers reported concerns about reduced organizational efficiency and flexibility, and some employees reported unintended consequences, such as increased message overload during work hours. This highlights the need to design disconnection strategies that are flexible, context-sensitive and co-created with users.

### Supervisor-directed programs

Only one study targeted supervisors. Heissler et al (35) evaluated a supervisor training program designed to reduce ambiguity around after-hours availability expectations. In surveys conducted before and six weeks after the training, employees reported an increase in explicit availability agreements and reduced ambiguity. However, there were no significant changes in perceived availability expectations, psychological detachment, emotional exhaustion, ICT-communication satisfaction, or work–life balance. These findings suggest that while a brief supervisor training program can help clarify norms, it may be insufficient on its own to improve implicit norms, employee health and well-being outcomes.

### Individual level employee-focused programs

In total, seven publications evaluated programs targeted towards employees. We divide these into an informational connectivity program (N=1), ICT-efficiency and availability training (N=1) and multi-component boundary management programs (N=5).

First, Pfaffinger et al (36) conducted an RCT testing an informational intervention aimed at promoting healthier connectivity behaviors. They found no improvements for any outcome. Notably, the intervention group experienced increased general stress compared to the control group, suggesting potential unintended consequences of a low-intensity awareness effort.

Second, Edvinsson et al (37) evaluated a combined individual- and group level workplace intervention targeting efficient ICT-use, work-nonwork boundaries and availability expectations in a quasi-experimental pre- and post-test study. While no overall effects were found, the intervention reduced the need for recovery in employees who had a high need for recovery at the start of the study.

Finally, five studies tested broader interventions addressing after-hours connectivity as a part of a wider boundary management program. Reinke & Ohly (38) evaluated an online self-training program (*Switch off and recharge*) in an RCT study. The program had a significant effect on seven of the eight examined outcomes: perceived learning, boundary control and enactment, work-related ICT use, psychological detachment, work–life balance, and negative affect.

Althammer et al (39–41) evaluated the *FlexAbility* program aimed to help workers cope with challenges of flexible work designs in two RCT. They reported improvements in psychological detachment, work–life balance, boundary management, positive affect and reduced emotional exhaustion, across the four-week follow-up time. They also reported that improved self-regulation partially explained the increase in positive affect and engagement.

Rich et al (42) evaluated a group-based intervention for physicians focusing on integrating self-care, digital boundary strategies (eg, availability management) and peer-support to increase general and digital well-being in a pre- and post-test trial. The program reduced disengagement and exhaustion and improved boundary control, but had no effect on general well-being. Follow-up interviews identified implementation enablers (eg, peer support, goal settings, written commitments) and barriers (eg, shift patterns, time constraints and fear of missing out on work-matters during leisure time). Participants also called for more practical guidance in implementing strategies.

### Study quality varied

The ten quantitative studies were assessed with the EPHPP tool (30) (see supplementary table S2). While all studies used valid and reliable outcome measures, study quality was rated as weak overall, primarily due to selection bias (eg, unclear recruitment or low response rates), lack of confounder adjustment, absence of blinding (in RCT), and high attrition from pre- to post-test. Three qualitative studies were assessed using the JBI checklist for qualitative research (see supplementary table S3. Overall, the study quality was moderate. Two studies had discrepancy between aim and method (not being able to measure the effect of law or regulations on productivity (31) or effectivity (34).

## Discussion

This systematic review synthesized evidence from 12 studies on interventions to reduce availability expectations and work-related connectivity outside work hours. The evidence base is still small and heterogenous. We identified (i) evidence gaps and weak study designs; (ii) the need for stronger implementation and cultural support for policy interventions, (iii) the central role of norms and managerial practices, (iv) the promise of multi-component and flexible strategies; and (v) the importance of person–environment fit for interventions' effectiveness.

### Evidence gaps and weak study designs limit confidence in conclusions

Across studies, methodological quality was generally low. Most used non-experimental designs, small samples, short follow-up periods, and self-reported outcomes. Only some of the individual-focused studies employed RCT, limiting causal inference regarding the effectiveness of interventions and policies. These limitations reduce confidence in the observed effects and suggest that the field is still at an early stage of development. However, although RCT are considered the gold standard for evaluating intervention effectiveness, they are not always feasible or appropriate, particularly for national-level policies. In such contexts, quasi-experimental and natural experiments may provide the strongest available evidence. For future national-level policies, if feasible, pre- and post-study designs combined with in-depth qualitative assessments of user experiences (eg, barriers of use), could substantially strengthen the evidence base. Future research would benefit from more rigorous study designs with well-powered samples.

**Table 3:** Evidence map synthesizing all findings according to their level and outcome types. [○=improved; □=worsened; ◊=not significant.]

Level	Author	After-hours availability				Wellbeing								Work-life				Job				ICT			
		Availability agreement	Availability ambiguity	Availability expectations		Detachment	Burnout	Positive affect	Negative affect	Stress	Need for recovery	Wellbeing		Boundary management	Work-life balance	Work-family conflict		Organizational commitment	Work engagement	Job satisfaction		Technostress	Satisfaction with ICT communication	Digitalization anxiety	IT resilience
Organizational	Barber et al, 2023																					◊			
Organizational	Mueller & Kempen, 2023						○											○	□						
Supervisor	Heissler et al, 2023	○	○	◊		◊	◊								◊								◊		
Employee	Althammer et al, 2023					○						○			○										
Employee	Althammer et al, 2024						○	○						○	○										
Employee	Althammer et al, 2025					○				○		○				○			○						
Employee	Edvinsson et al, 2025					◊					◊														
Employee	Pfaffinger et al, 2023					◊				□									◊	◊		◊		◊	◊
Employee	Reinke, 2024					○		◊	○					○	○										
Employee	Rich et al, 2020						○					◊		○											

### Policy interventions require stronger implementation and workplace support

Despite the growing attention towards right-to-disconnect efforts, both workplace disconnection guidelines and national right-to-disconnect legislation were underrepresented in the literature, with mixed findings. Disconnection policies were for instance not associated with lower telepressure (32). A study by Mueller & Kempen (33), however, found that the associations between disconnection policies and outcomes appeared contingent on employee–policy fit. Specifically, employees with strong preference for segmentation, a clear separation between work and personal life, experienced more favorable outcomes from disconnection policies than those preferring integration. Similarly, both qualitative and quantitative findings showed that disconnection policies had side-effects, such as the loss of flexibility and subsequent email overload (32, 34). These findings underscore the importance for organizations to consider the person–environment fit as well as general workload, when designing and implementing formal disconnection guidelines.

At the national level, only one study – on the French right-to-disconnect legislation – examined a legal disconnection policy. The findings indicated minimal impact, with most employees reporting no changes resulting from the policy (31). The legislation in France is intended to be flexible and context-dependent, allowing organizations to define and tailor their own internal practices and guidelines. While this approach potentially allows for a beneficial customization based on organizational and employee needs, it may also result in no or superficial implementation, thus undermining the intention of the law. In contexts with strong availability norms, top-down regulatory interventions, such as national disconnection laws may be likely to face resistance or remain unenforced (18). Without active organizational commitment, norm changes and supportive cultural norms, policy measures alone are unlikely to achieve meaningful change. This is in line with prior findings from the work–family research field, which also shows that formal policies, benefits or regulations alone tend to have limited consequences for employees. Their effectiveness depends on informal norms and managerial support [eg, Allen (43)]. In other words, explicit rules often matter less than the workgroup culture which will shape whether employees feel able to use them.

### Norms and managerial practices offer a novel venue of change

Although availability expectations are consistently linked to strain, poor recovery and work–life conflict (2, 11), only three interventions directly addressed availability expectations (35–37). Informational campaigns alone were ineffective or even counterproductive (36). Interventions targeting supervisors' availability expectations was associated with lower ambiguity and increased likelihood to have explicit availability agreements but was not associated with employees' health and well-being (35). Edvinsson et al's intervention (37) targeting efficient ICT-use, boundary management and improving availability expectations did not have a direct impact on employees' health. Limited duration and intensity of the interventions might however partly explain these null findings.

Recent experimental work has revealed a managerial “detachment paradox”, where managers recognize the benefits of detachment, but still often penalize employees who set boundaries (44). In line with this, Barber et al (32) found that while explicit norms (disconnection policies) were not associated with reduced telepressure, implicit norms (availability expectations and family-supportive work environment) were associated with reduced telepressure. These findings underscore the importance of targeting social and cultural drivers of connectivity, not just formal guidelines and policies.

Taken together, recent experimental work and the current evidence base of included studies suggest that targeting availability expectations is a critical, yet underdeveloped, avenue for intervention. Future research should develop and test theoretically grounded, multi-level interventions that actively challenge availability norms and incorporate disconnection strategies. Multi-level approaches, embedding clear expectations into routine organizational practice, such as onboarding of managers and employees, performance feedback and team discussions, may offer a more sustainable route to positive changes. The “optional work availability” framework (19) offers one promising, though still untested, example.

### Multi-component and flexible strategies show some promise

Some broader interventions involving disconnection components within broader multi-faceted programs targeting individuals, demonstrated positive outcomes (38–42). These programs often combined boundary management training with specific disconnection strategies, and some allowed tailoring to individual preferences and needs. The program flexibility may have enhanced the relevance and uptake of the program. However, it is difficult to disentangle which specific components were effective in these programs. Future studies should therefore test different component combinations to identify which elements are most promising.

### Effectiveness depends on person–environment fit

A consistent theme across studies was that associations and effects depended on individual preferences and circumstances. Strategies such as disconnection policies or fixed communication curfews might improve work–life balance for some employees but create new challenges for others, including subsequent email overload or reduced flexibility (34). Strict implementations or regulations, such server shutdowns or blocking email delivery at predefined hours, are viewed by many organizations as effective in limiting after-hours intrusion (1). However, as Pansu (31) highlights, while workers appreciate protection from intrusion, they also value flexibility. Rigid rules may inadvertently reduce autonomy and create stress if employees are unable to respond when necessary. Employees with caregiving responsibility or low segmentation preferences may find such measures particularly restrictive (33, 42). Moreover, employees might have to compress already heavy workloads into shorter time frames which could generate even more work stress. These patterns align with the person–environment fit theory (45), which posits that compatibility between individuals and their work context predicts satisfaction, commitment, and well-being. This means uniform disconnection rules could align with some employees' preferences but run counter to others as found in one of the included studies by Müller & Kempen (33). Future studies should thus not only assess general consequences of interventions for the general population but also explore moderators, such as boundary management preferences, work demands and caregiving responsibilities, which might camouflage effects. These findings align with broader occupational health research emphasizing the importance of tailoring interventions to individual and contextual needs. Moreover, it might be challenging to implement regulations if they are too strict and not seen as acceptable or feasible by the workers themselves. As such, participatory or co-design approaches, which actively involve employees in shaping interventions, may be essential to ensure fit and reduce unintended consequences.

### Strengths and limitations of the included studies

The current evidence base offers initial valuable insights into a range of efforts and policies aimed at reducing availability expectations and after-hours connectivity. However, the evidence base is still small and heterogenous, making it difficult to compare findings across studies. The included studies were also limited by small sample sizes, short follow-up periods, and a predominance of studies from high-income countries. There is also a lack of RCT designs and few studies directly assessed availability expectations and after-hours work connectivity despite this being a focus in the interventions. More diverse, methodologically rigorous and controlled longitudinal assessments are needed to strengthen the field. Future studies should be especially attentive and add resources already in the recruitment process of their study to ensure a high response rate as the risk of selection bias in the current pool of papers is rated as high. Second, studies should ensure that the intervention and control groups are similar or aim to describe to which degree these groups are different. Also, the association between availability expectations and connectivity and occupational stress might be bi-directional, with occupational stress increasing connectivity and vice versa. Where reverse causation from strain to connectivity is prominent, efforts targeting the causes of stress, rather than the connectivity may be more effective.

Third, researchers should make sure that blinding procedures are used and described when conducting controlled studies. The blinding should be conducted so that the researchers are not aware of the intervention status of the participant when conducting analyses. For instance, allocation to the different groups should be concealed with labels (group A versus B) so researchers analyzing the data do not know who was in the intervention versus the control group, and cues in the data that can reveal the status of the participants should be deleted (eg, time stamps if data is conducted at different time points). Fourth, researchers should make efforts to prevent high drop-out rates from pre- to post-test in follow-up studies. Moreover, few of the quantitative studies reported effect sizes, which are important to know for potential cost-benefit analyses.

We encourage future intervention studies across a broader range of countries and cultural contexts. To assess whether strategies are effective across cultural and policy contexts, the findings from high-income countries (ie, Germany, UK and Sweden) may not be globally generalizable. Broader evidence can support more context-sensitive and effective interventions. Particularly since the negative consequences of availability expectations and after-hours connectivity have been documented globally, prompting widespread debate and concern.

### Strengths and limitations of the current review

The current systematic review utilized a transparent and rigorous systematic approach to screen and quality assess publications according to predefined criteria. Nevertheless, there are limitations that need to be addressed. First, due to the heterogeneity of the studies, spanning different interventions, methods and outcomes, generalization across the included studies was challenging and we could not conduct a meta-analysis. Future reviews should update the search when the number of published studies has increased and consider conducting meta-analyses. Second, we did not include legal science papers as the inclusion criteria were requiring empirical data of employees or employers and it was out of the scope for the current paper. We do however acknowledge that law science studies can offer a further understanding of the implementation and potential efficacy of the right-to-disconnect, such as Bell et al (46) and Lerouge et al (22).

### Implications for policy and practice

Increased availability expectations and excessive after-hour connectivity are consistently associated with increased risk of adverse occupational health outcomes, such as stress, burnout, absenteeism, and work–life conflict. However, evidence on effective interventions remains limited, particularly at the workplace and national policy level. Our findings suggest that reducing harmful after-hours connectivity requires more than formal policies. It requires sustained organizational commitment, active norm-setting, and flexible, context-sensitive implementation. Without such efforts, policies and guidelines are unlikely to be effective, and right-to-disconnect legislation risk becoming largely symbolic. Legislative measures should therefore be accompanied by strategies that promote cultural change and organizational ownership. Employers should ensure that guidelines are actively integrated through leadership practices and team norms, while allowing flexibility to accommodate differing needs and preferences. Future well-designed intervention studies are needed to guide effective policy and practice.

## Supplementary material

Supplementary material
