# Supplementary material for: What do we know about limiting after-hours availability expectations and work-related connectivity? A systematic review of interventions and policies
Source: Scand J Work Environ Health. 2026 Apr 30;52(3):206–18. doi: 10.5271/sjweh.4277 (PMC13147545; doi:10.5271/sjweh.4277)
Supplement: Supplementary material [file SJWEH-52-206-S001.pdf]

What do we know about limiting after-hours availability expectations and work-related connectivity? A systematic review of interventions and policies[1]

by Wendy Nilsen, PhD,[2] Tanja Nordberg, PhD, Kristine Lescoeur, MSc, Mari Holm Ingelsrud, PhD, Cathrine Egeland, PhD

1. Supplementary material
2. Correspondence to: Wendy Nilsen, PhD, Work Research Institute / Arbeidsforskningsinstituttet, OsloMet, P.O. Box 4 St. Olavs plass, N-0130 Oslo, Norway. [E-mail: wendy.nilsen@oslomet.no] ORCID: 0000-0001-6178-0002.

Supplementary table 1: Systematic search by database and cluster, conducted 10.10.2024

	Cluster 1: Work connectivity	Cluster 2: Workplace	Cluster 3: Intervention	Cluster 1 AND Cluster 2 AND Cluster 3
Web of Science	90.833	7.989.263	2.584.043	1.251
Medline (Ovid)	49.780	3.046.985	1.604.990	382
PsychINFO (Ovid)	5.164	1.087.534	280.596	107
Embase (Ovid)	70.761	4.351.302	2.163.743	609
Total including duplicates				2349
Deleting duplicates				628
**Total**				**1721**

Notes: Date: All searches were conducted 10.10.2024 Time limits: 2004 to current date. Language limits: English language.

Supplementary table 2: Search string and findings in APA PsycInfo conducted in OVID database 10.10.2024

#	Query	Results
1	("business*" or "employee*" or "employer*" or "employment" or "job*" or "labor" or "labour" or "occupation*" or "office*" or "organization*" or "organisation*" or "work*" or "industr*" or "profession*").mp. [mp=title, abstract, heading word, table of contents, key concepts, original title, tests & measures, mesh word]	1,637,747
2	limit 1 to (english language and yr="2004 -Current")	1,087,534
3	("trial*" or "intervention*" or "RCT" or "experiment*" or "process evaluation" or "effectiveness" or "implement*" or "feasibility" or "acceptability" or "evaluation" or "prevent*" or "guideline*" or "policy" or "policies" or "guideline*" or "tactics" or "regulation*").m_titl.	413,482
4	limit 3 to (english language and yr="2004 -Current")	280,596
5	("right to disconnect" or "right-to-disconnect" or "right-to-disconnect" or "disconnect from work" or "digital detox" or "detach*" or "work connectivity" or "work-related connectivity" or "after-hours connectivity" or "after-hours communication" or "after-hours work" or "working after hours" or "off-job" or "outside work hours" or "after-work hours" or "off-hours" or "non-working hours" or "technology-assisted supplementary work" or "technology-assisted supplemental	7,994

	work" or "technology-assisted work" or "always on" or "always-on" or "job recovery" or "intensive connectivity" or "intensive smartphone" or "constant availability" or "job availability" or "availability demands" or "availability expectations" or "extended work availability" or "constant connectivity" or "boundary crossing ICT-use" or "boundary management" or "border management" or "work-related ICT use" or "work-related smartphone use" or "work-related technology use").mp. [mp=title, abstract, heading word, table of contents, key concepts, original title, tests & measures, mesh word]	
6	limit 5 to (english language and yr="2004 -Current")	5,164
7	2 and 4 and 6	107

Supplementary table 3: Search strings and findings in Embase conducted in OVID database 10.10.2024

#	Query	Results
1	("business*" or "employee*" or "employer*" or "employment" or "job*" or "labor" or "labour" or "occupation*" or "office*" or "organization*" or "organisation*" or "work*" or "industr*" or "profession*").mp. [mp=title, abstract, heading word, drug trade name, original title, device manufacturer, drug manufacturer, device trade name, keyword heading word, floating subheading word, candidate term word]	5,854,332
2	limit 1 to (english language and yr="2004 -Current")	4,351,302
3	("trial*" or "intervention*" or "RCT" or "experiment*" or "process evaluation" or "effectiveness" or "implement*" or "feasibility" or "acceptability" or "evaluation" or "prevent*" or "guideline*" or "policy" or "policies" or "guideline*" or "tactics" or "regulation*").m_titl.	3,044,463
4	limit 3 to (english language and yr="2004 -Current")	2,163,743
5	("right to disconnect" or "right-to-disconnect" or "right-to-disconnect" or "disconnect from work" or "digital detox" or "detach*" or "work connectivity" or "work-related connectivity" or "after-hours connectivity" or "after-hours communication" or "after-hours work" or "working after hours" or "off-job" or "outside work hours" or "after-work hours" or "off-hours" or "non-working hours" or "technology-assisted supplementary work" or “technology-assisted supplemental work” or "technology-assisted work" or "always on" or "always-on" or "job recovery" or "intensive connectivity" or "intensive smartphone" or "constant availability" or "job availability" or "availability demands" or "availability expectations" or "extended work availability" or "constant connectivity" or "boundary crossing ICT-use" or "boundary management" or "border management" or "work-related ICT use" or "work-related smartphone use" or "work-related technology use").mp. [mp=title, abstract, heading word, drug trade name, original title, device manufacturer, drug manufacturer, device trade name, keyword heading word, floating subheading word, candidate term word]	104,402
6	limit 5 to (english language and yr="2004 -Current")	70,761
7	2 and 4 and 6	609

Supplementary table 4: Search string and findings in Medline conducted in OVID database 10.10.2024

#	Query	Results
1	("business*" or "employee*" or "employer*" or "employment" or "job*" or "labor" or "labour" or "occupation*" or "office*" or "organization*" or "organisation*" or "work*" or "industr*" or "profession*").mp. [mp=title, book title, abstract, original title, name of substance word, subject heading word, floating sub-heading word, keyword heading word, organism supplementary concept word, protocol supplementary concept word, rare disease supplementary concept word, unique identifier, synonyms, population supplementary concept word, anatomy supplementary concept word]	4,426,998
2	limit 1 to (english language and yr="2004 -Current")	3,046,985
3	("trial*" or "intervention*" or "RCT" or "experiment*" or "process evaluation" or "effectiveness" or "implement*" or "feasibility" or "acceptability" or "evaluation" or "prevent*" or "guideline*" or "policy" or "policies" or "guideline*" or "tactics" or "regulation*").m_titl.	2,503,379
4	limit 3 to (english language and yr="2004 -Current")	1,604,990
5	("right to disconnect" or "right-to-disconnect" or "right-to-disconnect" or "disconnect from work" or "digital detox" or "detach*" or "work connectivity" or "work-related connectivity" or "after-hours connectivity" or "after-hours communication" or "after-hours work" or "working after hours" or "off-job" or "outside work hours" or "after-work hours" or "off-hours" or "non-working hours" or "technology-assisted supplementary work" or "technology-assisted work" or "always on" or "always-on" or "job recovery" or "intensive connectivity" or "intensive smartphone" or "constant availability" or "job availability" or "availability demands" or "availability expectations" or "extended work availability" or "constant connectivity" or "boundary crossing ICT-use" or "boundary management" or "border management" or "work-related ICT use" or "work-related smartphone use" or "work-related technology use").mp. [mp=title, book title, abstract, original title, name of substance word, subject heading word, floating sub-heading word, keyword heading word, organism supplementary concept word, protocol supplementary concept word, rare disease supplementary concept word, unique identifier, synonyms, population supplementary concept word, anatomy supplementary concept word]	82,900
6	limit 5 to (english language and yr="2004 -Current")	49,780
7	2 and 4 and 6	382

Supplementary table 5: Search string and findings in Web of Science Core Collection conducted 10.10.2024

#	Clusters, all limted to english language and 2004-current year		Results
1	**Cluster 1: Work connectivity** “right to disconnect” OR “right-to-disconnect” OR “right-to-disconnect” OR “disconnect from work” OR “digital detox” OR “detach*” OR “work connectivity” OR “work-related connectivity” OR “after-hours connectivity” OR “after-hours communication” OR “after-hours work” OR “working after hours” OR “off-job” OR “outside work hours” OR “after-work hours” OR “off-hours” OR “non-working hours” OR “technology-assisted supplementary work” OR “technology-assisted supplemental work” OR “technology-assisted work” OR “always on” OR “always-on” OR “job recovery” OR “intensive connectivity” OR “intensive smartphone” OR “constant availability” OR “job availability” OR “availability demands” OR “availability expectations” OR “extended work availability” OR “constant connectivity” OR “boundary crossing ICT-use” OR “boundary management” OR “border management” OR “work-related ICT use” OR “work-related smartphone use” OR “work-related technology use”	Topic	90.833
2	**Cluster 2: Workplace** “business*” OR “employee*” OR “employer*” OR “employment” OR “job*” OR “labor” OR “labour” OR “occupation*” OR “office*” OR “organization*” OR “organisation*” OR “work*” OR “industr*” OR “profession*”	Topic	7.989.263
3	**Cluster 3: Intervention** “trial*” OR “intervention*” OR “RCT” OR “experiment*” OR "process evaluation" OR “effectiveness” OR “implement*” OR “feasibility” OR “acceptability” OR “evaluation” OR “prevent*” OR “guideline*” OR “policy” OR “policies” OR “guideline*” OR “tactics” OR “regulation*”	Title	2.584.043
4	1 AND 2 AND 3		1251

Supplementary Table 6: Quality assessment of studies based on EPHPP criteria: Subs cores and global rating

Author	Selection bias	Study design	Control of confounders	Blinding	Data collection	Withdrawals and dropouts	Total score (5-18)	Global rating
Althammer et al., 2023	Weak	Strong	Weak	Weak	Strong	Weak	14	Weak
Althammer et al., 2024	Weak	Strong	Strong	Weak	Strong	Weak	12	Weak
Althammer et al., 2025	Weak	Strong	Strong	Weak	Strong	Moderate	11	Weak
Barber et al., 2023	Moderate	Weak	Weak	N/A	Strong	N/A	9	Weak
Edvinsson et al., 2024	Weak	Moderate	Strong	Weak	Strong	Strong	11	Weak
Heissler et al., 2023	Moderate	Moderate	Weak	N/A	Strong	Weak	11	Weak
Mueller & Kempen, 2023	Weak	Weak	Strong	N/A	Strong	N/A	8	Weak
Pfaffinger et al., 2023	Weak	Strong	Strong	Moderate	Strong	Weak	11	Weak
Reinke, 2024	Weak	Strong	Weak	Weak	Strong	Moderate	13	Weak
Rich et al., 2020	Weak	Moderate	Weak	N/A	Strong	Strong	10	Weak

Note: Total score computed by converting strong = 3, moderate =2, weak = 1, and N/A = 0, and computing a sum score. Global rating was assigned according to the EPHPP guidelines (strong = no weak ratings, moderate = one weak rating, weak = two or more weak ratings).

Supplementary table 7: Qualitative studies assessed with The Joanna Briggs Institute (JBI) Critical Appraisal Checklist for Qualitative Research

Study	Q1	Q2	Q3	Q4	Q5	Q6	Q7	Q8	Q9	Q10	Score
Pansu et al., 2018	/	/	/	1	1	0	0	1	0	1	4/10
Reis et al.	1	1	1	1	1	0	0	1	0	1	7/10
Rich et al.	1	1	1	1	1	0	0	1	1	1	8/10

Note 1: Key: 1 = Yes, 0 = No, / = Unclear.
Note 2: Q1-Q10:

Q1.*Philosophy–method fit*
Q2.*Method–research question fit*
Q3.*Methodology–method fit*
Q4.*Method–analysis fit*
Q5.*Method–interpretation fit*
Q6.*Researcher position stated*
Q7.*Reflexivity addressed*
Q8.*Participants' voices represented*
Q9.*Ethics approval documented*
Q10. *Conclusions supported by data*

Percentage of "Yes" responses per question (across three studies):

Question	% Yes
Q1	66%
Q2	66%
Q3	66%
Q4	100%
Q5	100%
Q6	0%
Q7	0%
Q8	100%
Q9	33%
Q10	100%

Q1-Q10 in full text:

1. Is there congruity between the stated philosophical perspective and the research methodology?
2. Is there congruity between the research methodology and the research question or objectives?
3. Is there congruity between the research methodology and the methods used to collect data?
4. Is there congruity between the research methodology and the representation and analysis of data?
5. Is there congruity between the research methodology and the interpretation of results?
6. Is there a statement locating the researcher culturally or theoretically?
7. Is the influence of the researcher on the research, and vice- versa, addressed?
8. Are participants, and their voices, adequately represented?
9. Is the research ethical according to current criteria or, for recent studies, and is there evidence of ethical approval by an appropriate body?
10. Do the conclusions drawn in the research report flow from the analysis, or interpretation, of the data?
